# Mechanisms of resistance to EGFR-targeted therapies in colorectal cancer: more than just genetics

**DOI:** 10.3389/fcell.2023.1176657

**Published:** 2023-09-15

**Authors:** Christine Parseghian, Madhulika Eluri, Scott Kopetz, Kanwal Raghav

**Affiliations:** ^1^ Department of Gastrointestinal Medical Oncology, The University of Texas MD Anderson Cancer Center, Houston, TX, United States; ^2^ Division of Cancer Medicine, The University of Texas MD Anderson Cancer Center, Houston, TX, United States

**Keywords:** anti-EGFR, anti-EGFR rechallenge, genomic mechanisms of resistance to anti-EGFR, non-genomic mechanisms of resistance to anti-EGFR, anti-EGFR resistance

## Abstract

The development of acquired resistance to anti-EGFR therapies remains poorly understood, with most research to date exploring, and trying to overcome, various genomic mechanisms of resistance. However, recent work supports a model of resistance whereby transcriptomic mechanisms of resistance predominate in the presence of active cytotoxic chemotherapy combined with anti-EGFR therapy in the first-line setting, with a greater predominance of acquired MAPK mutations after single-agent anti-EGFR therapy in the later-line setting. The proposed model has implications for prospective studies evaluating anti-EGFR rechallenge strategies guided by acquired MAPK mutations and highlights the need to address transcriptional mechanisms of resistance.

## Highlights

• EGFR inhibitors are approved for the treatment of patients with *RAS* wild-type metastatic colorectal cancer in combination with chemotherapy or as a single-agent in the chemo-refractory setting; however, almost all patients develop resistance eventually.
• To date, resistance to EGFR inhibitors has been ascribed to acquisition of mutations in the MAPK pathway. However, these mutations are only seen in half of all patients, suggesting that additional mechanisms of resistance exist.
• Recent evidence evaluating circulating tumor DNA (ctDNA) has demonstrated that resistance mechanisms to EGFR inhibitors differ when they are used alone or when combined with chemotherapy, depending on line of therapy.
• Mechanistic underpinnings of this differential resistance pattern remain a key gap in our knowledge of EGFR targeted therapy.
• Novel clinical trial designs and pre-clinical studies exploring these gaps and demonstrating clinical benefit are necessary to better understand and ultimately translate these findings to the clinic.

## Introduction

Anti-EGFR therapies, specifically anti-EGFR monoclonal antibodies such as cetuximab and panitumumab, combined with chemotherapy have resulted in a survival benefit for patients with left-sided metastatic colorectal cancer (mCRC) with *RAS/RAF* wildtype tumors (Heinemann et al., 2014; Venook et al., 2017; Yoshino et al., 2022). However, almost all patients develop resistance to these therapies within 4–8 months of therapy initiation. The development of acquired resistance to anti-EGFR therapies remains poorly understood, with various genomic and non-genomic mechanisms postulated. While *KRAS*, *NRAS*, *BRAF*, *MAP2K1*, and *EGFR* ectodomain (EGFR-ECD) mutations have been shown to be a source of acquired resistance, they are only present in 40%–50% of cases leading to the question of additional mechanisms of resistance (Diaz et al., 2012; Misale et al., 2012; Montagut et al., 2012; Morelli et al., 2015; Van Emburgh et al., 2016; Pietrantonio et al., 2017; Parseghian et al., 2022). Circulating tumor DNA (ctDNA) has presented an opportunity to further understand both the temporal dimensionality and process of tumorigenesis and may help us understand the pathogenesis of resistance mechanisms to anti-EGFR and other targeted therapies (Montagut et al., 2018; Strickler et al., 2018).

Studies utilizing serial ctDNA samples have demonstrated that acquired *RAS* and *EGFR* ectodomain alterations decay exponentially with time, resulting in a half-life of approximately 4.4 months (Siravegna et al., 2015; Parseghian et al., 2019; Topham et al., 2022). This has led to anti-EGFR rechallenge as a therapeutic opportunity for RAS/RAF wildtype patients. This strategy has resulted in response rates of up to 30% when selecting patients without pre-existing *RAS* and *EGFR* alterations on ctDNA immediately prior to anti-EGFR re-challenge initiation (Cremolini et al., 2019; Martinelli et al., 2021; Sartore-Bianchi et al., 2022). While objective response rates and progression free-survival benefits appear promising in these studies, additional larger, randomized studies will be needed prior to routine clinical adoption, and the limited efficacy raises questions about possible non-genomic mechanisms of resistance.

Recently, two studies by our group utilizing paired ctDNA samples have demonstrated unique molecular patterns of resistance between first-line and later-line anti-EGFR therapies (Parseghian et al., 2023; Raghav et al., 2023). These observations have potential far-reaching consequences related to longitudinal ctDNA monitoring, implications for timing of anti-EGFR therapy, and potential diverging mechanisms of emerging resistance based on line of therapy.

## Differential of acquired mutations in front line *versus* second line

Conventional understanding that was starting to build with earlier evidence from retrospective studies was that resistance to anti-EGFR agents in mCRC is a result of acquired alterations in MAPK pathway, specifically acquired *KRAS*, *NRAS*, *BRAF*, *MAP2K1*, and *EGFR* ectodomain (EGFR-ECD) mutations and *ERBB2* and *MET* amplifications in a substantial subset (40%–50%) of the population exposed to these agents (Parseghian et al., 2022). However, most of these studies used anti-EGFR in later-line setting (after first-line setting). With the recent impetus on moving the anti-EGFR therapy to the upfront setting, especially in *RAS/BRAF*-WT left-sided mCRC (Venook et al., 2017), data emerging from resistance in the first line setting in combination with cytotoxic chemotherapy is fast emerging and sketching a vastly different profile.

We show in two retrospective ctDNA-based biomarker analyses of randomized cohorts (anti-EGFR with chemotherapy vs. chemotherapy (with/without anti-VEGF agent) that the frequency of acquired genomic resistance alterations in the MAPK pathway were substantially lower in the first line setting (6%–9%) compared to that seen with use of anti-EGFR in later lines (46%–62%). Furthermore, preclinical modeling also demonstrated that acquired resistance to either cetuximab or chemotherapy appears to be a result of cross-resistant transcriptomic profiles consistent with epithelial-to-mesenchymal transition (Parseghian et al., 2023).

## Increased passenger mutations with known acquired resistance mutations on anti-EGFR therapy

Conventionally, Darwinian selection has been the predominant hypothesis to rationalize the development of acquired resistance to targeted therapies. By this theory, resistance is generated by the progressive domination of pre-existing therapy-resistant subclones. However, an alternative hypothesis known as adaptive mutability has been proposed as a mechanism implicated in this progression (Russo et al., 2019). The adaptive mutability model hypothesizes a temporary switch from a high-fidelity to an error-prone mutagenic state under therapeutic stress. To test this hypothesis, we evaluated passenger mutation burden, defined as alterations other than *KRAS/NRAS/EGFR/BRAF/MAP2K1*, that may emerge with first-line vs. third-line anti-EGFR therapies (Parseghian et al., 2023). It was observed that passenger mutations were three-fold greater in the third-line setting (1.4 v 0.4 mutations, respectively; *p* < 0.001) and patients who had ctDNA evidence for acquired RAS and EGFR alterations were more likely to develop additional passenger mutations than those who did not (2.3 v 0.9, respectively; *p* < 0.001). These results suggest that there may be heterogenous patterns of acquired resistance related to chemotherapy combination and sequencing and have important implications for appropriate patient selection for anti-EGFR and other targeted therapies.

## Subclones of acquired mutations rarely expand

Concordantly, in both studies we observed that the frequency of acquired genomic resistance mutations was significantly higher in the third-line setting compared to the first line (62% v 6.6% and 46% v 9%, respectively). Further, Raghav et al. showed that the acquisition of genomic alterations between patients who received bevacizumab in combination with chemotherapy in the first-line setting was comparable to those that received anti-EGFR with chemotherapy. Similarly, Vidal and colleagues found that in five of 9 patients with *RAS/BRAF* resistance mutatations by Cycle 3, the RAS subclone did not necessarily expand. Both of these studies go against the Darwinian selection hypothesis; however, they do open the possibility of chemotherapy driving resistance in the first-line setting, even when combined with targeted therapies. Taken together, these studies suggest that chemotherapy-based multi-agent therapies may favor a single resistance profile that may include transcriptomic, epigenetic and tumor-microenvironment changes rather than resistance to single component of the regimen. Specifically, understanding how mechanisms of resistance differ between multi-drug regimens and single-agent therapies have implications in our ability to generate future successful combination therapies. Similar to initial chemotherapy regimens, it will be necessary when adding molecularly-targeted therapies to understand whether the combination is synergistic through cytotoxicity or in its ability to alter mechanisms of therapeutic resistance, or a combination of both. The basis of this understanding will be essential for the development of next-generation combinatorial approaches and their sequencing.

## Shared mechanisms of resistance between anti-EGFR and cytotoxic chemotherapy

We were also able to demonstrate the need to delineate mechanisms of resistance in the first-line metastatic setting (Parseghian et al., 2023). The significant difference in acquisition of mutations in the first-line setting with combination anti-EGFR and cytotoxic chemotherapy compared to the second and third-line setting, which have historically been the setting wherein anti-EGFR is introduced, suggests that selective pressure due to chemotherapy *versus* targeted therapies may evolve with time. Specifically, CMS2 subtype has been shown to have prolonged benefit to anti-EGFR; however, over time there may be a switch to a CMS4 subtype, which is associated with increased transforming growth factor beta (TGF-β) and an endothelial-to-mesenchymal (EMT) phenotype (Guinney et al., 2015).

In contrast to prior work suggesting that transcriptomic mechanisms of resistance may dominate with anti-EGFR monotherapy (Sadanandam et al., 2013; Guinney et al., 2015; Woolston et al., 2019), these studies suggest that non-genomic mechanisms of resistance may predominate when anti-EGFR therapy is combined with chemotherapy, as seen in the first-line studies compared to third-line (Parseghian et al., 2023; Raghav et al., 2023).

To further explore this, we conducted translational analyses evaluating cell lines with and without acquired cetuximab resistance showed both phenotypic and gene expression profiles consistent with EMT (Parseghian et al., 2023). Transforming growth factor beta (TGF- β) which is a defining characteristic of the CMS4 subtype was notably upregulated in the cetuximab-resistant cell line compared to the parental cell line. Additional confirmatory testing showed concomitant increase in E-cadherin, snail, slug and vimentin, proteins shown to have a critical role in EMT induction (Vu and Datta, 2017). Subsequently, to evaluate anti-EGFR efficacy after chemotherapy, several 5-fluorouracil, oxaliplatin, and SN38-resistant cell lines were treated with cetuximab and showed less sensitivity with prolonged anti-EGFR treatment. Though this *in vitro* data is limited, along with the genomic data described in the two studies, it suggests that CRC cells may develop resistance to chemotherapy and anti-EGFR through nongenomic mechanisms compared to later-lines when anti-EGFR is given as a single agent. Altogether this work portends the importance of expanding and confirming these findings prospectively and may result in therapeutic opportunities to prevent subtype switching.

## Discussion

Evidence regarding emergence of resistance mechanisms against anti-EGFR monoclonal antibodies in mCRC is evolving. Anti-EGFR rechallenge with and without ctDNA monitoring has been clinically adopted (Sartore-Bianchi et al., 2022) in mCRC. About a third of select patients with tissue *RAS* WT tumors who have had prior benefit from and then subsequently progressed on anti-EGFR therapy-based regimen in any line, who have no acquired *RAS*, *BRAF* and *EGFR ECD* mutations in ctDNA, can have response to anti-EGFR rechallenge. However, patient selection and ctDNA selection can be augmented by understanding the true nature of resistance to anti-EGFR agents, subject to the line of therapy and combinatorial partner. Though promising, the generalizability of these results remains limited by small sample sizes. In order to translate these results to the clinic, additional prospective evidence through large-scale clinical trials and mechanistic studies are needed to both better understand and develop novel strategies to overcome the underpinnings of these genomic and nongenomic resistance mechanisms.

## Data Availability

The original contributions presented in the study are included in the article/supplementary material, further inquiries can be directed to the corresponding author.
